# Enhancing biomass and lipid accumulation in the microalgae *Schizochytrium* sp. by addition of fulvic acid and EDTA

**DOI:** 10.1186/s13568-018-0681-5

**Published:** 2018-09-21

**Authors:** Xiao-Man Sun, Lu-Jing Ren, Xiao-Jun Ji, He Huang

**Affiliations:** 1 College of Biotechnology and Pharmaceutical Engineering, Nanjing Tech University, No. 30 South Puzhu Road, Nanjing, 211816 People’s Republic of China; 2 School of Pharmaceutical Sciences, Nanjing Tech University, No. 30 South Puzhu Road, Nanjing, 211816 People’s Republic of China; 3 State Key Laboratory of Materials-Oriented Chemical Engineering, Nanjing Tech University, No. 5 Xinmofan Road, Nanjing, 210009 People’s Republic of China; 4 Jiangsu National Synergetic Innovation Center for Advanced Materials (SICAM), Nanjing, People’s Republic of China

**Keywords:** *Schizochytrium* sp., Fulvic acid, EDTA, Lipid, Oxidative stress

## Abstract

Enhancing lipid productivity and reducing oxidative damage is essential for lipid overproduction in microalgae. In this study, addition of 20 mg/L fulvic acid (FA) resulted a 34.4% increase of lipid yield in *Schizochytrium* sp. Furthermore, the cooperative effect of FA and EDTA on cell growth and lipid production was investigated. The combined addition of 20 mg/L FA and 1.0 g/L EDTA yielded a maximal cell dry weight of 130.7 g/L and lipid productivity of 1.16 g/L/h, representing 36.4% and threefold increase over the non-supplemented group, respectively. Moreover, compared with the non-supplemented group, the combined addition strategy exhibited overall lower levels of reactive oxygen species and malondialdehyde, which accompanied with 66.7% and 81.9% higher superoxide dismutase and catalase activity, respectively. Furthermore, a 24.1–37.1% increase of malic enzyme and 19.4–25.2% decrease of phosphoenolpyruvate carboxylase activity was observed during the entire fermentation stage (0–108 h). Results suggested that the combined addition strategy not only enhanced lipid accumulation, but also prevented the lipid peroxidation.

## Introduction

Microalgae have received growing interest as a potential biofuel feedstock, and are regarded as a promising alternative source for next-generation renewable fuels (Bellou et al. 2014). Microalgal lipids are classified into two types according to their carbon number, with fatty acids containing 14–20 carbons used for biodiesel production, and polyunsaturated fatty acids (PUFA) with more than 20 carbon atoms used as health food supplements. As a heterotrophic oleaginous microalga, *Schizochytrium* sp. can accumulate over 50% of lipids with a high fraction of PUFA in its cell dry weight (Ling et al. 2015; Zhao et al. 2018). It consequently received increasing attention as a promising feedstock for the sustainable production of biofuels and food (Markou and Nerantzis 2013). However, cost-effective and biocompatible cultivation strategies for lipid production in *Schizochytrium* sp. need to be explored further.

Previous studies mainly focus on improving the fermentation process to enhance the lipid content of *Schizochytrium* sp., and the stress-based strategy is considered as the most successful inducing method (Sahin et al. 2018). However, cellular responses to the stress-based strategy, including low biomass and reactive oxygen species (ROS) formation, are the two major negative effects. The resultant ROS can react with biomacromolecules (such as DNA, lipid and protein) and result in damage, thus leading to loss of protein function and even cell death (Ruenwai et al. 2015). The general countermeasure is to adopt the two-stage culture strategy, apply the optimal growth conditions at the first stage into maximizing the biomass production, and preserve the lipid accumulation at the second stage. For example, the final lipid yield of *Nannochloropsis oculata* obtained in a two-stage process was 2.82-times higher than that of single-stage cultivation systems (Su et al. 2011). Moreover, many recent studies improved microalgae growth lipid accumulation by reduction of oxidative stress by developing high stress tolerant strain and exogenous addition of growth antioxidants. For instance, adaptive evolution under cooperative low temperature and high salinity conditions obtained mutants of *Schizochytrium* sp. with strong stress tolerance and increased accumulation of lipid rich in PUFA (Sun et al. 2018). In another study, overexpression of superoxide dismutase (SOD) in *Schizochytrium* sp. also successfully alleviated oxidative stress and increased the PUFA content by 32.9% (Zhang et al. 2018). Apart from strain development, the addition of antioxidants offers a simple way to alleviate cellular oxidative damage. For instance, the lipid yield and docosahexaenoic acid (DHA) productivity was increased by 14.5% and 20.0% in *Schizochytrium* sp. and *Crypthecodinium cohnii* by adding ascorbic acid and sesamol, respectively (Liu et al. 2015; Ren et al. 2016). However, the primary effect of antioxidants was only to reduce oxidative damage, rather than induce lipid biosynthesis itself.

It has been reported that plant hormones not only reduce the oxidative damage in cells but also increase the lipid production by controlling internal biochemical pathways (Lu and Xu 2015), which presents new opportunities for improving microalgal lipid production. As a plant growth regulator, fulvic acid (FA) plays a key role in controlling hormone level and improving the secondary metabolite level (Çimrin et al. 2010). For example, the biomass of *Haematococcus pluvialis* was increased by 7.13% and 10.58% after the addition of 5 and 10 mg/L FA, respectively (Zhao et al. 2015). Moreover, the highest lipid content can be achieved in *Monoraphidium* sp. FXY-10 by exogenous addition of 120 mg/L FA, which is 1.8 times higher compared with the control treatment (Che et al. 2016a). It has been known that ethylene diamine tetraacetic acid (EDTA) is the common component in microalgae growth medium, which can increase the cell permeability through inducing the membrane pore. In recent years, supplementation of EDTA has been also reported to improve the lipid accumulation in microalgae. In *Nannochloropsis oculata*, the accumulation of biomass and lipids gradually increased with increased concentrations of EDTA (Dou et al. 2013). Moreover, Ren et al. (2014) had also reported that the lipid content in *Scenedesmus* sp. was markedly increased from 50 to 45% when the EDTA concentration is increased from 0 to 0.001 g/L. However, previous studies were focused only on a single phytohormone or EDTA when evaluating their effects on lipid accumulation. Moreover, the precise mechanisms of the improvement of lipid accumulation by exogenous application of phytohormones or EDTA remain elusive.

In this study, the cooperative effect of FA and EDTA on cell growth, lipid productivity, and lipid profile was investigated in *Schizochytrium* sp. Furthermore, the activities of antioxidant defenses, four key enzymes involved in lipid biosynthesis, as well as the levels of oxidative damage indicators including ROS and MDA, were determined to explore the mechanisms guiding the physiological and molecular changes triggered by FA and EDTA. The aim of this study was to develop a promising strategy to improve lipid productivity, which also might be used in other oil-producing microalgae.

## Materials and methods

### Microorganism and culture conditions

*Schizochytrium* sp. HX-308 used in this study was isolated from the sea water, which stored in the China Center for Type Cultuer Collection (Number: CCTCC M 209059). The strain was preserved in 20% (v/v) glycerinum at − 80 °C. The seed medium and breeding conditions were the same as those used in our previous study (Qu et al. 2011). After three cell passages, the seed culture (1% v/v) was transferred into the 500 mL shake flask containing 100 mL medium for shaking culture in the constant tract at 30 °C at 170 rpm. The obtained cell suspension was used as the inoculant for fermentation at inoculum size of 10% (v/v). The fermentation medium was the same as that reported in our previous study (Ren et al. 2010). The basic medium were dissolved in artificial sea water, which contained 50 g/L glucose and 20 g/L monosodium glutamate. One liter of artificial sea water contained (g/L): 0.1 g CaCl_2_, 10 g Na_2_SO_4_, 4 g KH_2_PO_4_, 0.8 g (NH4)_2_SO_4_, 2 g MgSO_4_, 0.2 g KCl and the trance elements: 0.6 g CuSO_4_·5H_2_O, 0.29 g FeSO_4_, 0.8 g ZnSO_4_, 0.01 g Na_2_MoO_4_·2H_2_O, 0.01 g CoCl_2_·6H_2_O, 0.06 g NiSO_4_·6H_2_O and 0.86 g MnCl_2_·4H_2_O.

### Determination of cell dry weight and total lipids

Ten milliliter (mL) culture aliquot was obtained after 5 min of centrifugation at 4500×*g*, which was used to determine the cell dry weight through gravimetric analysis. Subsequently, the cells were transferred onto the weighing filter paper and dried at 60 °C to constant weight (about 12 h). Meanwhile, the total lipids, extracellular glucose and glutamic acid contents were determined according to the methods used in our previous study (Qu et al. 2011).

### Fatty acid analysis

Fatty acid methyl ester (FAME) was prepared from 0.2 g dry cells and analyzed using the GC-2010 gas chromatography system (Shimadzu, Japan) equipped with the DB-23 capillary column (60 m × 0.22 mm; Agilent, USA). Meanwhile, the flame ionization detector (FID) was also employed in this study. Nitrogen was used as the carrier gas. The injection port was maintained at 250 °C, with the injection volume of 1 μL. The column temperature was increased from 100 to 200 °C at a rate of 2 °C/min, and to 230 °C at a rate of 4 °C/min and then maintained for 9 min. Temperature of the FID was set at 280 °C. Specifically, FAME was identified through comparing the retention time with the corresponding external true reference standard (Sigma, USA). Besides, nonane-decanoid acid (C19:0) was used as the internal standard to estimate the individual FAME content based on the integral peak area in the chromatogram.

### Preparation of cell extracts and enzyme assay

Microorganisms were obtained through centrifugation, washed with the icy distilled water, and washed with the washing buffer [400 mM Tris–HCl buffer, pH 7.4, containing 20% (w/v) glycerinum and 1 mM dithiothreitol (DTT)]. Finally, the microorganisms were suspended in the clean washing buffer supplemented with 0.5 mM phenyl-methane-sulfonyl fluoride (PMSF). The material was placed in the ultrasonic knapper for 15 min of crushing, and centrifuged at 4 °C for 15 min at 13,800×*g*, and the supernatant was immediately collected to determine the enzyme activity. Besides, protein was also determined according to the Bradford method using bovine serum albumin (BSA) as the standard. Afterwards, the total protein content (μg/mL) in the crude enzyme was calculated according to the empirical calibration equation (protein content = 0.1833 OD_595 nm_ − 0.0033).

Malic enzyme (ME) and glucose-6-phosphate dehydrogenase (G6PDH) activities was determined spectrophotometrically by monitoring the rate of NADPH formation at 28 °C and 25 °C, respectively. SOD and catalase (CAT) activities were determined using an assay kit (Nanjing Jiancheng Bioengineering Institute, China) according to the manufacturer’s instructions. The enzyme activity unit (U) was defined as the quantitative formation of NADPH/min, which equaled to 0.001/min increase at OD_340 nm_. The specific activity (U/mg protein) was defined as the activity unit/mg protein.

### Determination of intracellular reactive oxygen species and lipid peroxidation

The types of ROS were determined in accordance with the same methods reported in our previous study (Ren et al. 2016). According to the method proposed by Heath and Packer (1968), the malondialdehyde (MDA) equivalent was measured to determine the lipid peroxidation level. Additionally, the treated and untreated cells were homogenized in 5% (w/v) TCA and centrifuged at 10,000×*g* for 10 min at 4 °C. The reaction mixture containing the thiobarbituric acid-TCA solution and cell extracts were boiled for 20 min; later, the absorbance of the reaction mixture was recorded at 532 and 600 nm. The absorbance value recorded at 532 nm was subtracted by the non-specific absorbance value recorded at 600 nm, and the MDA equivalent content was measured using the 155 mM/cm extinction coefficient.

## Results

### Effect of FA on biomass and lipid production

Addition of FA at various concentrations (0, 10, 15, 20, 25 and 30 mg/L) into the medium would result in markedly changes in the cell growth and lipid accumulation of *Schizochytrium* sp. As shown in Table 1, the increase in FA concentration had given rise to the remarkable increases in cell dry weight (CDW) and lipid production. The optimal FA concentration was 20 mg/L, and the maximum CDW at that concentration was 47.7 g/L, while the lipid production was 16.4 g/L, which were 23.3% and 34.4% higher than those in the non-supplementation group, respectively. Such results were consistent with those reported in previous studies, namely, FA had the potential to enhance the lipid production in *Monoraphidium* sp. FXY-10 (Che et al. 2016b). Moreover, FA can increase the tolerance of microalgae to biotic and abiotic stresses, which might contribute to better cell growth and counteract the drop of microalgal productivity during prolonged fermentation. The maximum lipid productivity of 0.41 g/L/h was obtained in the group supplemented with 20 mg/L FA. However, a further increase in FA concentration to 25 and 30 mg/L resulted in a drop of CDW and lipid productivity, suggested that high FA concentrations (over 20 mg/L) might actually hinder the growth of *Schizochytrium* sp. The biochemical basis remained unclear, but previous studies had verified that, high concentration of plant growth regulator 2,4-dichlorophenoxyacetic acid would also suppress cell proliferation (Saygideger and Okkay 2008).

**Table 1:** Cell growth and lipid production of *Schizochytrium* sp. under different concentrations of fulvic acid

	FA concentration (mg/L)					
	0	10	15	20	25	30
CDW (g/L)	38.7 ± 0.7	39.9 ± 0.6	42.4 ± 0.8	47.7 ± 0.5	40.5 ± 0.7	34.2 ± 0.5
Lipid yield (g/L)	12.2 ± 0.3	12.5 ± 0.2	12.9 ± 0.1	16.4 ± 0.3	13.6 ± 0.4	9.7 ± 0.3
Fermentation time (h)	45	42	40	40	45	50
Lipid productivity (g/L/h)	0.27	0.30	0.32	0.41	0.30	0.19

Data represent the mean values and standard deviations of three replicates for each measurement

### Cumulative effects of FA and EDTA on biomass and lipid production

In order to enhance the availability of FA or nutrients to *Schizochytrium* sp., EDTA was added into the medium in concentrations ranging from 0.1 to 2.0 g/L in addition to the supplementation with 20 mg/L FA (Table 2). At EDTA concentrations from 0.1 to 1.0 g/L, the lipid yield increased along with a higher lipid productivity of 0.52 g/L/h, representing 26.8% and 92.6% increases over the supplementation of 20 mg/L FA without EDTA supplemented and no supplementation whatsoever, respectively. However, further increase in EDTA concentration to 2.0 g/L could also dramatically suppress microalgae growth, and the final lipid production rate was only 0.35 g/L/h upon the end of the experiment. High EDTA concentration was well-known to strongly chelate metal ions (Kern et al. 1992), suggesting that high EDTA level would distinctly reduce the availability of metal ions essential for the growth of microalgae, leading to low total lipid production. Similarly, Ren et al. (2014) had reported that, further increase in EDTA concentration to 1.0 g/L would apparently restrain the growth of microalgae *Scenedesmus* sp. Therefore, the addition of 20 mg/L FA and 1.0 g/L EDTA was the most promising strategy for lipid accumulation in *Schizochytrium* sp.

**Table 2:** Cell growth and lipid production of *Schizochytrium* sp. under different concentrations of EDTA on the premise of adding 20 mg/L fulvic acid

	20 mg/L FA + EDTA concentration (g/L)				
	0.1	0.2	0.5	1.0	2.0
CDW (g/L)	47.2 ± 0.3	46.8 ± 0.6	50.1 ± 0.4	51.4 ± 0.3	46.5 ± 0.5
Lipid yield (g/L)	16.2 ± 0.7	17.6 ± 0.3	18.8 ± 0.6	20.6 ± 0.5	15.8 ± 0.4
Fermentation time (h)	40	40	40	40	45
Lipid productivity (g/L/h)	0.41	0.44	0.47	0.52	0.35

Data represent the mean values and standard deviations of three replicates for each measurement

### The batch-fermentation behavior of *Schizochytrium* sp. with the combined addition strategy

**Fig. 1:**
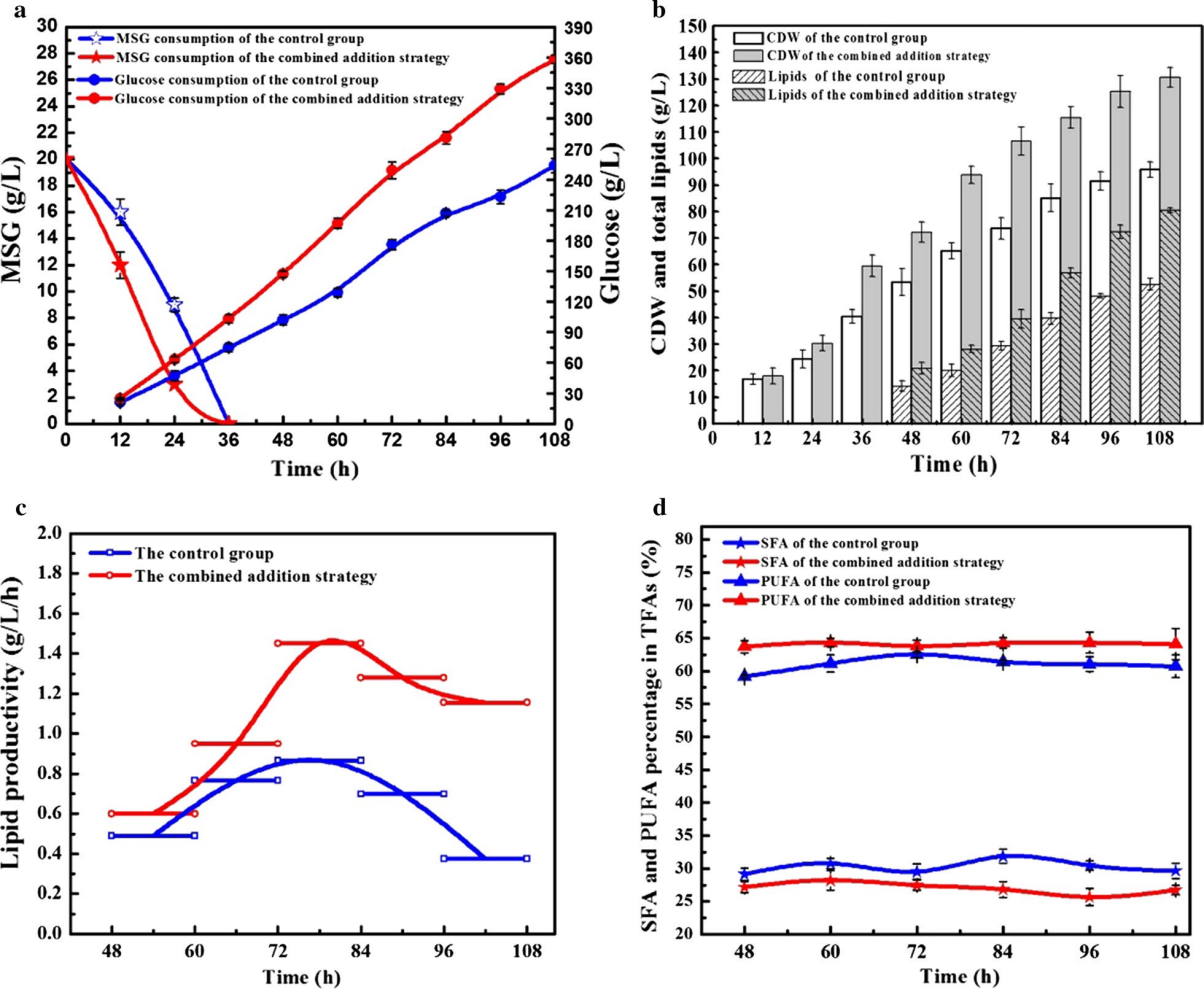
Comparison between the control group and the combined addition strategy in a 5-L bioreactor: **a** substrate consumption, **b** CDW and lipid content, **c** lipid productivity, **d** SFA and PUFA percentage in TFAs. Values and error bars represent the means and standard deviations from triplicate experiments

To further investigate the practicability of the combined addition strategy (20 mg/L FA and 1.0 g/L EDTA) in the production of lipids, we used it in conjunction with conventional culture in a 5-L bioreactor. As shown in Fig. 1a, a significant difference in glucose consumption was identified between the non-supplemented cultures and those supplemented with 20 mg/L FA and 1.0 g/L EDTA. Compared to the control group, *Schizochytrium* sp. exhibited faster substrate consumption with the combined addition strategy, which might be attributed to higher cell permeability induced by EDTA. Moreover, FA can also improve cell growth by controlling internal biochemical pathways. The cooperative effect of two chemicals resulted the highest CDW of 130.7 g/L at the end of the fermentation, which was 36.4% higher than that of the control group (Fig. 1b).

The total lipid yield increased sharply in both groups after nitrogen exhaustion (36 h). A maximum yield corresponding to 80.4 g/L of total lipids was obtained with the combined addition strategy, which was 52.9% higher than that of the control group (Table 3). In has been reported that FA can increase lipid production by enhancing the degradation of carbohydrates to biosynthesis lipids (Che et al. 2016b), which is consistent with the higher glucose consumption observed with the combined addition strategy. Moreover, as shown in Fig. 1c, the lipid productivity increased with culture time in both groups, reached its maximum at 84 h and then declined at later fermentation stages. Compared with the control group, the combined addition group exhibited an overall higher lipid productivity during the entire fermentation period, especially during the later fermentation stage (> 72 h). At the end of fermentation, the lipid productivity reached 1.16 g/L/h with the combined addition strategy, which was about threefold higher than that of the control group. These results were in contrast with several reports on the application of other plant growth regulators, such as jasmonic acid and indole-3-acetic acid, which induced lipid accumulation only during the early growth phase (Jusoh et al. 2015).

**Table 3:** Fermentation results of the combined strategy in a 5 L bioreactor and % changes compared to the same results from the control group

Parameters	The control group	The combined addition strategy	Increase (%)
CDW (g/L)	95.8 ± 2.8	130.7 ± 3.7	36.4
Lipid yield (g/L)	52.6 ± 2.3	80.4 ± 0.9	52.9
Lipid in CDW (w/w)	0.55	0.62	12.7
Lipid productivity (g/L/h)	0.49	0.74	51.0
SFA percentage (% TFAs)	29.7 ± 1.2	26.8 ± 0.7	− 9.8
PUFA percentage (% TFAs)	60.7 ± 1.7	64.1 ± 2.4	5.6

The control group: no-addition of FA and EDTA; the combined addition strategy: addition of 20 mg/L FA and 1.0 g/L EDTA. Data represent the mean values and standard deviations of three replicates for each measurement

Interestingly, addition of FA and EDTA would not only markedly affect cell growth and lipid production, but would also significantly influence the fatty acid composition. The saturated fatty acid (SFA) in *Schizochytriu*m sp. was mainly composed of C14:0 and C16:0, while the polyunsaturated fatty acid (PUFA) was mainly comprised of docosenoic acid (DPA) and docosahexaenoic acid (DHA). As shown in Fig. 1d, the SFA and PUFA biosynthesis values were inversely related in both cultures. Notably, the total fatty acid (TFA) and SFA percentages in the cultures supplemented with FA and EDTA were always lower than those in the control. By contrast, the PUFA percentage in TFAs increased in cultures supplemented with 20 mg/L FA and 1.0 g/L EDTA, resulting in 64.1% PUFA in TFAs. Moreover, a decline of the PUFA percentage was observed in the control group after 60 h. PUFA are well- recognized antioxidants, and a large number of studies had been carried out focusing on their production and use (Richard et al. 2008). After 60 h, *Schizochytrium* sp. might begin to consume the PUFA to protect itself from oxidative injury, and such secondary consumption might account for the reduction in PUFA percentage. Accordingly, the combined addition strategy safeguarded an overall better PUFA percentage, which implied that FA and EDTA can alleviate the oxidative damage typical for later fermentation stages.

### Influence of the combined addition strategy on the anti-oxidative defense systems for *Schizochytrium* sp.

**Fig. 2:**
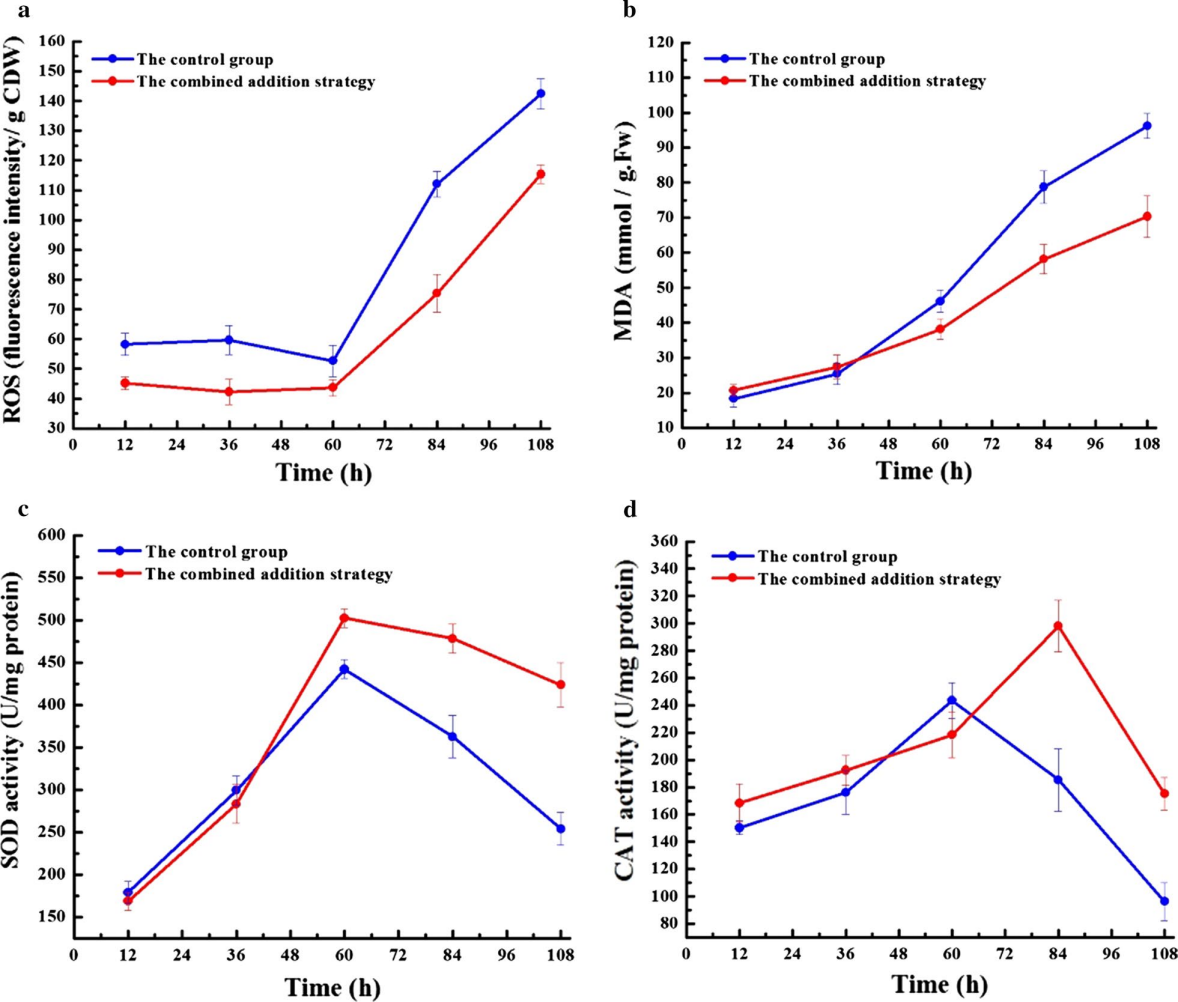
Comparison of anti-oxidative defense system between the control group and the combined addition strategy in a 5-L bioreactor: **a** ROS levels, **b** MDA levels, **c** SOD activity, **d** CAT activity. Values and error bars represent the means and standard deviations from triplicate experiments

In order to investigate the effects of FA and EDTA on the oxidative defense systems of *Schizochytrium* sp., the concentrations of ROS and MDA, as well as the activities of antioxidant enzymes were determined. Cellular ROS mainly include the superoxide anion (O_2_^−^), hydrogen peroxide (H_2_O_2_), hydroxyl radical (·OH), lipid hydroperoxides (LOOH), and peroxyl radicals (LOO·) (Zhang et al. 2017). During the initial stage (< 60 h), *Schizochytrium* sp. cells were in the exponential growth phase and were able to produce ample antioxidants to resist oxidative stress. Consequently, both cultures maintained low ROS levels during this stage (Fig. 2a). Moreover, lipid peroxidation is another commonly used stress maker, which can be measured through detecting the MDA content in cell (Heath et al. 1968). Similar to ROS levels, the MDA levels also sharply increased in both cultures after 60 h (Fig. 2b). Conversely, the addition of FA and EDTA reduced the ROS and MDA levels, especially after 60 h. The ROS levels in the supplemented group were reduced by 22.5% at 12 h and remained significantly lower at 108 h compared to the non-supplemented control group. By the end of fermentation, a minimum MDA level of 70.3 mmol/g Fw was measured in the culture using the combined addition strategy, which was 26.9% lower than that of the control group (Fig. 2b). In contrast, an overall strong SOD and CAT activity was observed in the culture utilizing the combined addition strategy (Fig. 2c, d). For example, by the end of fermentation, SOD and CAT activity reached up to 423.5 and 175.2 U/mg protein, which was 66.7% and 81.9% higher than that of the control group, respectively (Fig. 2c, d).

### Influence of the combined addition strategy on the activities of key enzymes of *Schizochytrium* sp.

**Fig. 3:**
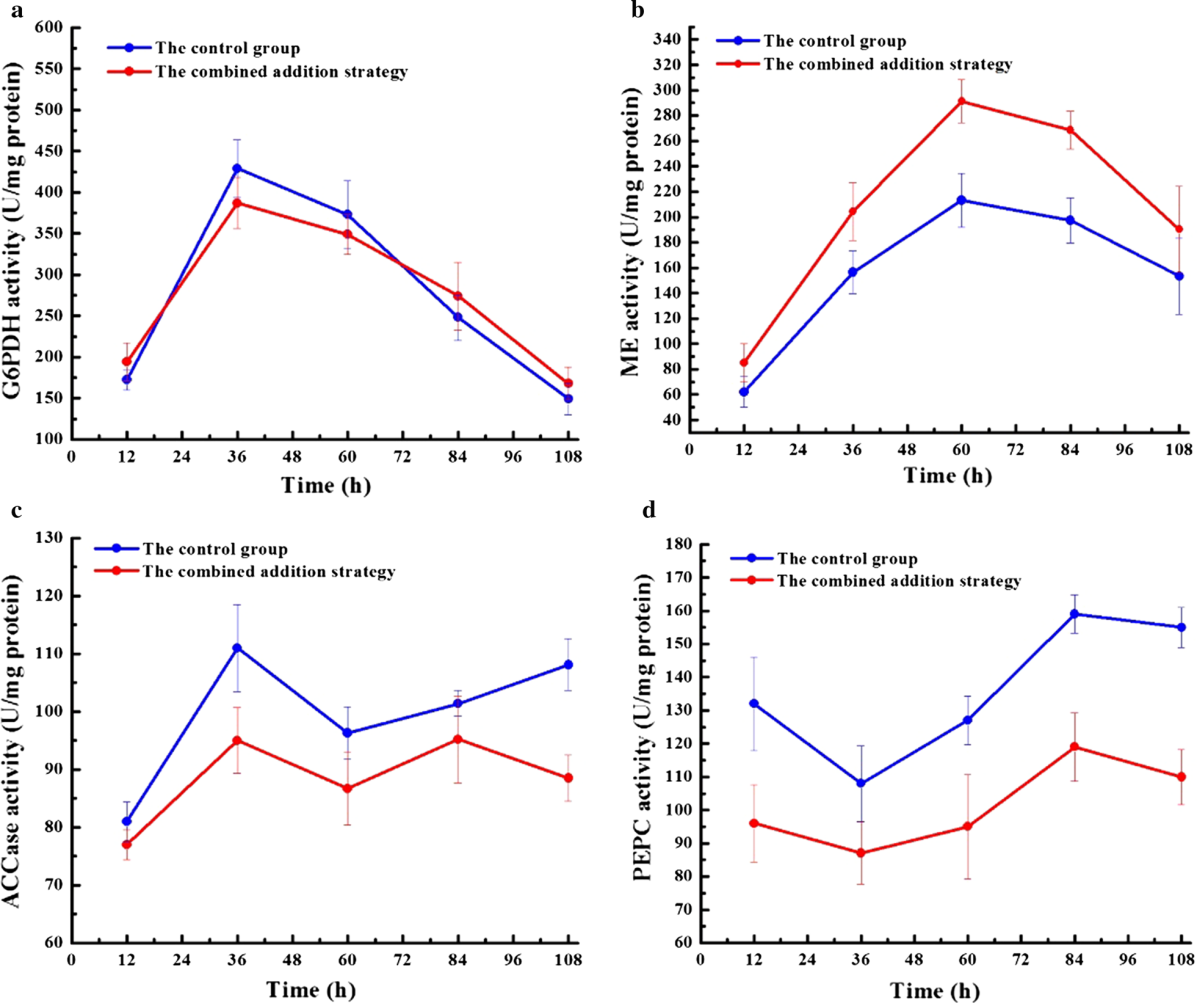
Comparison of key enzymes involved lipid biosynthesis between the control group and the combined addition strategy in a 5-L bioreactor: **a** G6PDH activity, **b** ME activity, **c** ACCase activity, **d** PEPC activity. Values and error bars represent the means and standard deviations from triplicate experiments

In order to further investigate the regulatory mechanisms mediating the observed effects of FA and EDTA on lipid production, the activities of key enzymes related to lipid accumulation (G6PDH, ME, ACCase, and PEPC) were detected every 24 h during the entire fermentation process. As shown in Fig. 3a, the activity of G6PDH increased in both cultures with increasing culture time and reached its maximum at 36 h, with no obvious differences. However, the combined addition strategy led to an overall higher ME activity than that of the control group. The ME activity reached 291.3 U/mg protein at 60 h, representing an increase of 36.6% over the control (Fig. 3b). In addition, the time point of the maximum value of ME activity (60 h) lagged far behind the time point of highest G6PDH activity (36 h). This illustrates that G6PDH is responsible for the NADPH supply during the early fermentation stages, while ME mainly functions to resolve the insufficiency of NADPH supply during the later stages.

Moreover, the addition of FA and EDTA resulted in a reduction of ACCase activity to 88.5 U/mg protein at 108 h, which was 18.1% lower than the corresponding value of the control group (Fig. 3c). One of the ROS species, the hydroxyl radical (·OH), can react with bicarbonate to form an intermediate bicarbonate radical, which is a crucial step needed at the active site of the ACCase enzyme (Menon et al. 2013). In this study, the lower ROS levels might have decreased the ACCase activity in the culture with the combined addition strategy. Similarly, compared to the control group, a 19.4–25.2% decrease of PEPC activity was observed with the combined addition strategy during the entire fermentation process (Fig. 3d).

## Discussion

As a kind of aerobiotic microalgae, ROS production is an inevitable aspect for *Schizochytrium* sp. Moreover, lipids, especially PUFA, can be oxidized themselves (Niki 2009), and the lipid peroxidation can also lead to ROS accumulation at a high level. However, microalgae has evolved an adaptive approach to eliminate and neutralize the ROS; for instance, the application of a variety of antioxidant enzymes. Therefore, it is important to control the balance between oxidative injury and antioxidant ability in *Schizochytrium* sp. for lipid accumulation. In fact, nitrogen starvation can induce oxidative damage to cells, but this negative effect on exponential phase is greatly lower than stationary phase, because cells at exponential phase can product lots of stress factors to alleviate the oxidative damage (Liu et al. 2012). As shown in Fig. 1a, nitrogen (MSG) was depleted at 36 h, but the ROS levels was decreased at 36–60 h (Fig. 2a), which might attribute to the production of antioxidants by *Schizochytrium* sp. After 60 h, with the rapid accumulation of lipid, lipid peroxidation could result in high level of ROS till the end of fermentation (Ruenwai et al. 2015). During this stage, cells begin the experience oxidative stress, since the amount of ROS has exceeded the antidotic capacity of the antioxidant defense system. ROS accumulation can induce oxidative injury to the cellular components, and exert adverse influence on cell growth, which may explain for the low CDW and the reduced PUFA percentage in the non-supplemented group (Fig. 1b, d).

Nonetheless, during the whole fermentation process, relatively low ROS level while high SOD and CAT activities are maintained in the supplemented group (Fig. 2c, d), suggesting that the combined addition strategy can mitigate oxidative injury through enhancing the activity of antioxidant enzymes. Similarly, some evidence indicates that plant hormone can regulate the oxidative stress response in microalgae (Han et al. 2018). For instance, the addition of abscisic acid can greatly reduce the oxidative stress in *Chlamydomonas reinhardtii* through enhancing the CAT and APX activities. Consistently, in *Chlorella vulgaris*, the greatest enhancements in antioxidant activities (55% for SOD, 89% for CAT, and 75% for APX) can be attributed to the addition of indol-3-acetic acid (Piotrowska-Niczyporuk and Bajguz 2014). Actually, plant hormone is generally related to the ROS-induced signal transduction. For example, the mitogen-activated protein kinase (MAPK)-activated H_2_O_2_ treatment can down-regulate the expression of auxin response gene (Kovtun et al. 2000). Recently, Beck et al. (2007) had proposed that abscisic acid could participate in the ROS signaling pathway, and regulated ROS production and clearance, which might be a potential oxidative stress regulatory mechanism. Interestingly, the MDA level is markedly reduced in the combined addition strategy, demonstrating that FA and EDTA can not only enhance lipid accumulation, but can also prevent lipid loss resulted from peroxidation.

As is well known, the reducing energy provided in the form of NADPH plays a key role in lipid biosynthesis (Tan and Lee 2016), while the transhydrogenase circulation and pentose phosphate pathway (PPP) are the two major NADPH regeneration pathways in *Schizochytrium* sp. Of them, G6PDH and ME are the respective key enzymes. In this study, the combined addition strategy has resulted in the overall higher ME activity than that in the control group, while G6PDH activity is not markedly changed (Fig. 3a, b). These results indicate that, compared with ME, G6PDH only plays a mild role in the lipid accumulated NADPH supply. Such phenomenon can also be observed in the microalgae *Aurantiochytrium* sp. (Song et al. 2013). Interestingly, the latest research indicates that, the PPP pathways can bind with the polyketide synthase (PKS) to provide NADPH for the biosynthesis of PUFA, whereas the transhydrogenase system can couple with the FAS pathway to provide NADPH for SFA biosynthesis (Beopoulos et al. 2011; Liang and Jiang 2015). Over-expression of G6PDH gene in *Aurantiochytrium* sp can successfully improve the PUFA percentage by 10.6% (Cui et al. 2016). However, overexpression of G6PDH gene in *Phaeodactylum tricornutum* would lead to reduction in PUFA proportion by about 22.7%, although the lipid content is increased by 2.7 times (Xue et al. 2017). Such difference may be attributed to the fact that *Aurantiochytrium* sp has synthesized PUFA through the PKS pathway, while *Phaeodactylum tricornutum* has adopted the desaturase/elongase pathway. In *Schizochytrium* sp., the FAS enzyme complex is of crucial importance to the synthesis of SFA, while the PKS pathway is responsible for PUFA synthesis (Ren et al. 2010). In this study, the SFA percentage is not increased, but the combined addition strategy has increased the ME activity, indicating that FA and EDTA can more effectively produce the total lipids, rather than change the fatty acid profile. In recent years, many studies used chemical regulators to improve biomass and lipid accumulation of thraustochytrids. In the previous study, the CDW and lipid yield of *Schizochytrium* sp. can be increased to 106.7 g/L and 65.5 g/L after addition of 9 g/L of ascorbic acid, which was 16.2% and 30.4% higher than that of the non-supplemented group, respectively (Ren et al. 2016). Moreover, in *Aurantiochytrium* sp. YLH70, addition of 4 mg/mL of gibberellin resulted in the maximum biomass of 21.4 g/L and lipid yield of 11.6 g/L, representing a 14.4% and 43.6% higher than that of the non-supplemented group, respectively (Yu et al. 2016).

Acetyl-CoA has been well recognized to be the major precursor for fatty acid biosynthesis, while the acetyl-CoA carboxylase (ACCase) and PEPC are the key enzymes related to the carbon precursor supply. ACCase can catalyze the first step of fatty acid biosynthesis, namely, the transformation of acetyl-CoA to malonyl-CoA, which is also the rate-limiting step of fatty acid biosynthesis (Davis et al. 2000). In this study, the combined addition strategy has restricted the ACCase activity, which is theoretically contradictory to the high lipid productivity. However, it is found in another study that, addition of FA can increase the ACCase activity by three times in *Monoraphidium* sp. FXY-10 (Che et al. 2016b) suggests that the action of FA depends on the microalgae species. Actually, almost no attempt to improve ACCase activity to improve lipid productivity is successful. For instance, in *Nannochloropsis oceanica* and *Neochloris oleoabundans*, the expression of ACCase gene is greatly reduced under nitrogen deprivation, but the total lipid content is outstandingly increased (Li and Xu 2014; Rismani-Yazdi et al. 2012). PEPC can irreversibly catalyze the carboxylation of phosphoenolpyruvic acid into oxaloacetic acid, and introduce the metabolic flux into the Krebs circulation, which can provide the substrates and energy for cell growth, thus reducing the carbon flux towards lipid biosynthesis (Tian et al. 2014). Consequently, suppressing PEPC to promote the carbon flux towards fatty acid biosynthesis can promote lipid production, which is consistent with the reduced PEPC activity observed in this study (Fig. 3d). Such result is highly consistent with that in another study (Che et al. 2016b). As a result, PEPC knockdown in *Chlamydomonas reinhardtii* and *Thalassiosira pseudonana* can increase the TAG level by 74.4% and four times, respectively (Trentacoste et al. 2013; Kao and Ng 2017).

In conclusion, the strategy of adding 20 mg/L FA and 1.0 g/L EDTA is developed in this study to induce lipid biosynthesis and reduce oxidative injury. Meanwhile, the effects of the combined strategy on cell growth, lipid production, and lipid profile were investigated. Results indicated that cell growth and lipid yield were sharply increased by 36.4% and 52.9%, respectively. These major performance changes were accompanying with the reduced ROS level and enhanced antioxidant enzyme activities, proving that the combined addition strategy can effectively alleviate cell oxidative injury. Moreover, ME activity was considerably increased, whereas PEPC activity was significantly decreased. These findings have illustrated the potential molecular changes for the excessive lipid production related to the combined addition strategy.

## Abbreviations

ACCase: acetyl-CoA carboxylase
CAT: catalase
CDW: cell dry weight
EDTA: ethylenediaminetetraacetic acid
FA: fulvic acid
PEPC: phosphoenolpyruvate carboxylase
PUFA: polyunsaturated fatty acids
ROS: reactive oxygen species
SFA: saturated fatty acids
SOD: superoxide dismutase
